# 377. Phase I trial of two novel SARS-CoV-2 beta variant receptor-binding domain recombinant protein and mRNA vaccines as 4th dose boosters

**DOI:** 10.1093/ofid/ofad500.447

**Published:** 2023-11-27

**Authors:** Terry Nolan, Georgia Deliyannis, Sabine Braat, Lilith Allen, Jennifer Audsley, Marcin Ciula, Michelle Giles, Maryanne Griffith, Nicholas Gherardin, Tom Gordon, Samantha Grimley, Lana Horng, David Jackson, Jennifer Juno, Katherine Kedzierska, Stephen Kent, Sharon Lewin, Mason Littlejohn, Hayley McQuilten, Francesca Mordant, Oanh Nguyen, Vanessa Pac Soo, Briony Price, Damian Purcell, Pradhipa Ramanathan, Steven Rockman, Zheng Ruan, Joseph Sasadeusz, Julie Simpson, Kanta Subbarao, Chee Wah Tan, Joseph Torresi, Jing Jing Wang, Linfa Wang, Harry Al Wassiti, Chinn Yi Wong, Colin Pouton, Dale Godfrey

**Affiliations:** The Peter Doherty Institute for Infection and Immunity, Murdoch Children’s Research Institute, Melbourne, Victoria, Australia; Doherty Institute, Melbourne, Victoria, Australia; University of Melbourne, Melbourne, Victoria, Australia; Doherty Institute, Melbourne, Victoria, Australia; Doherty Institute, Melbourne, Victoria, Australia; Doherty Institute, Melbourne, Victoria, Australia; Doherty Institute, Melbourne, Victoria, Australia; Doherty Institute, Melbourne, Victoria, Australia; Doherty Institute, Melbourne, Victoria, Australia; Flinders University, Adelaide, South Australia, Australia; Doherty Institute, Melbourne, Victoria, Australia; Doherty Institute, Melbourne, Victoria, Australia; Doherty Institute, Melbourne, Victoria, Australia; Doherty Institute, Melbourne, Victoria, Australia; Doherty Institute, Melbourne, Victoria, Australia; Doherty Institute, Melbourne, Victoria, Australia; Doherty Institute, Melbourne, Victoria, Australia; Doherty Institute, Melbourne, Victoria, Australia; Doherty Institute, Melbourne, Victoria, Australia; Doherty Institute, Melbourne, Victoria, Australia; Doherty Institute, Melbourne, Victoria, Australia; Doherty Institute, Melbourne, Victoria, Australia; Doherty Institute, Melbourne, Victoria, Australia; Doherty Institute, Melbourne, Victoria, Australia; Doherty Institute, Melbourne, Victoria, Australia; CSL Seqirus, Melbourne, Victoria, Australia; Doherty Institute, Melbourne, Victoria, Australia; Doherty Institute, Melbourne, Victoria, Australia; University of Melbourne, Melbourne, Victoria, Australia; Doherty Institute, Melbourne, Victoria, Australia; Duke-NUS, Singapore, Not Applicable, Singapore; Doherty Institute, Melbourne, Victoria, Australia; Flinders University, Adelaide, South Australia, Australia; Duke-NUS, Singapore, Not Applicable, Singapore; Monash University, Melbourne, Victoria, Australia; Doherty Institute, Melbourne, Victoria, Australia; Monash University, Melbourne, Victoria, Australia; Doherty Institute, Melbourne, Victoria, Australia

## Abstract

**Background:**

SARS-CoV-2 booster vaccination needs to enhance protection against variants and minimise immune imprinting. The beta variant drove broad immunity against other SARS-CoV-2 variants, including omicron. We developed 2 vaccines targeting the beta variant receptor-binding domain (RBD): a recombinant dimeric RBD-human IgG_1_F_c_ -fusion protein, and an mRNA encoding a membrane-anchored RBD in a novel lipid nanoparticle.

**Methods:**

76 healthy adults aged 18–64y, previously vaccinated with 3 doses of licensed SARS-CoV-2 vaccines, were randomised to receive a 4^th^ dose of either an adjuvanted (MF59^®^, CSL Seqirus) protein vaccine (5, 15 or 45µg, N=32), or mRNA vaccine (10, 20, or 50µg, N=32), or placebo (saline, N=12) at least 90 days after 3^rd^boost or prior COVID infection (Fig 1). All participants received one dose of study vaccine or placebo on Day 1, in double-blind manner. Bleeds occurred on days 1 (prior to vaccination), 8, 29, 90 and 180, and safety monitoring was conducted for 180d. An external comparison group of healthy adults who received a 4^th^ dose booster of a licensed bivalent mRNA COVID vaccine were evaluated for immunogenicity. ClinicalTrials.gov NCT05272605.

**Figure 1**

**Figure:**
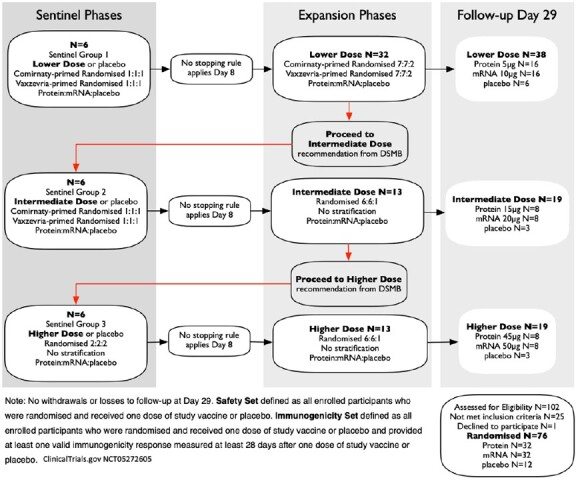

Trial profile

**Results:**

No vaccine-related serious or medically-attended AEs occurred. Protein vaccine reactogenicity profile was mild (no Grade-3). The mRNA was slightly more reactogenic at higher dose levels. Optimal anti-RBD antibody responses were recorded for the 45µg dose of protein vaccine and for 50µg of mRNA vaccine, but titre fold rise (GMFR) was stronger for the lower mRNA dose (Fig 2). A similar pattern was seen with live virus neutralisation and surrogate & pseudovirus neutralisation, including against BQ.1.1 and XBB.1.5 subvariants (Fig 3). Binding antibody titres were stronger for both study vaccines compared to those from a licensed bivalent mRNA COVID vaccine (Fig 2). T-cell studies showed a balanced Th1-Th2 profile, with CD4 & CD8 activation by both vaccines, stronger for CD8 with the mRNA vaccine.

**Fig 2.**

**Figure:**
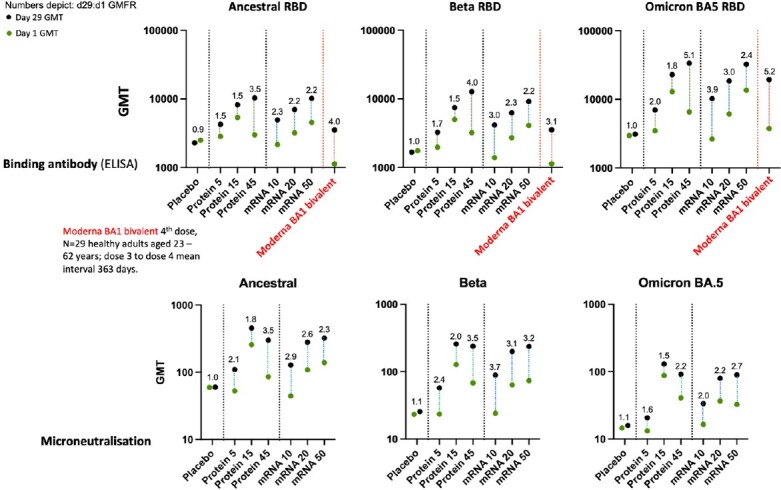

Pre- (Day 1) and Post-booster (Day 29) Immunogenicity: Binding antibody (ELISA) and Microneutralisation.

**Fig 3.**

**Figure:**
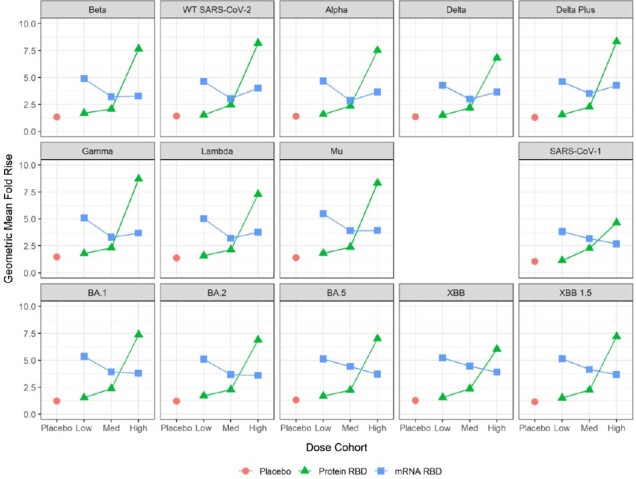

Booster seroresponses: Geometric mean fold rises (GMFR) by vaccine type and dose, surrogate virus neutralisation (sVNT).

**Conclusion:**

Both protein and mRNA beta RBD vaccines showed strong immune boosting against beta, ancestral and omicron strains, and was similar to that of a licensed bivalent mRNA COVID vaccine. There were no safety concerns and the reactogenicity profile was mild.

**Disclosures:**

**Terry Nolan, MD, PhD**, Clover: Board Member|CSL Seqirus: Advisor/Consultant|CSL Seqirus: Grant/Research Support|Dynavax: Grant/Research Support|GSK: Advisor/Consultant|GSK: Board Member|GSK: Grant/Research Support|Iliad: Grant/Research Support|Moderna: Advisor/Consultant|Moderna: Grant/Research Support|MSD: Advisor/Consultant|MSD: Grant/Research Support|Novavax: Board Member|Pfizer: Advisor/Consultant|Sanofi: Advisor/Consultant|Sanofi: Grant/Research Support|SK Bio: Board Member **Sharon Lewin, MBBS PhD**, Abbvi: Advisor/Consultant|Esfam: Advisor/Consultant|Gilead: Advisor/Consultant|Gilead: Honoraria|MSD: Honoraria|Vaxxinity: Advisor/Consultant|VIIV: Advisor/Consultant **Steven Rockman, PhD**, CSL Seqirus: employee|CSL Seqirus: Stocks/Bonds **Dale Godfrey, PhD**, CSL Seqirus: Grant/Research Support

